# Can Nrf2 Modulate the Development of Intestinal Fibrosis and Cancer in Inflammatory Bowel Disease?

**DOI:** 10.3390/ijms20164061

**Published:** 2019-08-20

**Authors:** Simona Pompili, Roberta Sferra, Eugenio Gaudio, Angelo Viscido, Giuseppe Frieri, Antonella Vetuschi, Giovanni Latella

**Affiliations:** 1 Department of Biotechnological and Applied Clinical Sciences, University of L’Aquila, 67100 L’Aquila, Italy; 2 Department of Anatomical, Histological, Forensic Medicine and Orthopedic Sciences, Sapienza University of Rome, 00185 Rome, Italy; 3 Department of Life, Health and Environmental Sciences, Gastroenterology, Hepatology and Nutrition Division, University of L’Aquila, 67100 L’Aquila, Italy

**Keywords:** inflammatory bowel disease, IBD, fibrosis, intestinal fibrosis, cancer, colorectal cancer, Nrf2

## Abstract

One of the main mechanisms carried out by the cells to counteract several forms of stress is the activation of the nuclear factor erythroid 2-related factor (Nrf2) signaling. Nrf2 signaling controls the expression of many genes through the binding of a specific *cis*-acting element known as the antioxidant response element (ARE). Activation of Nrf2/ARE signaling can mitigate several pathologic mechanisms associated with an autoimmune response, digestive and metabolic disorders, as well as respiratory, cardiovascular, and neurodegenerative diseases. Indeed, several studies have demonstrated that Nrf2 pathway plays a key role in inflammation and in cancer development in many organs, including the intestine. Nrf2 appears to be involved in inflammatory bowel disease (IBD), an immune-mediated chronic and disabling disease, with a high risk of developing intestinal fibrotic strictures and cancer. Currently, drugs able to increase cytoprotective Nrf2 function are in clinical trials or already being used in clinical practice to reduce the progression of some degenerative conditions. The role of Nrf2 in cancer development and progression is controversial, and drugs able to inhibit abnormal levels of Nrf2 are also under investigation. The goal of this review is to analyze and discuss Nrf2-dependent signals in the initiation and progression of intestinal fibrosis and cancers occurring in IBD.

## 1. Introduction

Inflammatory bowel disease (IBD) is a spectrum of diseases, including Crohn’s disease (CD) and ulcerative colitis (UC), that lead to numerous complications, among which the most common is represented by intestinal fibrosis and cancer. Intestinal fibrosis is a process characterized by excessive deposition of extracellular matrix (ECM) proteins by activated myofibroblasts [1,2,3].

Intestinal fibrogenesis is driven by complex mechanisms since in the gut there are many cell types (as fibroblasts, subepithelial myofibroblasts, smooth muscle cells, epithelial and endothelial cells) that may become activated ECM-producing myofibroblasts. In fibrosis, the abnormal accumulation of ECM can be determined either by its excessive production by activated myofibroblasts or by its reduced degradation. Physiologically, the degradation of ECM components is regulated by the delicate equilibrium between proteolytic enzymes, like matrix metalloproteinases (MMPs) and tissue inhibitors of MMP (TIMPs). Perturbation of the delicate MMP/TIMP balance results in the excessive deposition of ECM proteins, which consequently leads to fibrosis.

A pivotal role in intestinal fibrosis, other than in many types of cancer, is certainly played by transforming growth factor β (TGF-β), not only through its canonical interaction with small mothers against decapentaplegic proteins (Smads) but also by a complex network with other profibrotic and antifibrotic molecules [1,4,5,6]. Recently, several studies have focused their attention on Nrf2, an important transcription factor able to interact with TGF-β and that appears to be involved in the fibrogenesis process in many organs, including the bowel [7,8,9,10,11].

Additionally, although the exact mechanism of action seems still controversial, it was established that Nrf2 participates in the development, progression, and metastasis of several tumors, including colorectal cancer [12,13,14,15,16,17,18].

Understanding the exact role of Nrf2 signaling in the pathophysiology of these diseases could be crucial in developing new effective drugs [19,20,21].

Among the multiple aspects of Nrf2, in this review, we discuss its role in intestinal fibrosis and in colorectal cancer occurring during IBD and the possibility that it may be a new and valid therapeutic target.

## 2. What Is Nrf2?

Nowadays, the human body is repeatedly exposed to many external stimuli, such as environmental pollens, food additives, drugs, ultraviolet light, and ionizing radiation. In addition to these exogenous stresses, cells are also subject to biological insults due to free radicals, reactive oxygen species (ROS), and reactive nitrogen species (RNS) [22,23,24].

Under normal conditions, these molecules are synthesized in mitochondria, peroxisomes, and endoplasmic reticulum at physiological concentrations, while during pathological processes, they are released at high levels into the vascular system, becoming toxic [24] and leading to specific dysfunctions such as inflammation, autoimmune reaction, and cancer, as well as cardiovascular, airway, digestive, metabolic, and neurodegenerative diseases [23]. In order to neutralize these harmful effects, cells have developed a wide range of defensive mechanisms, including the activation of Nrf2 [24,25,26].

**Figure 1:**
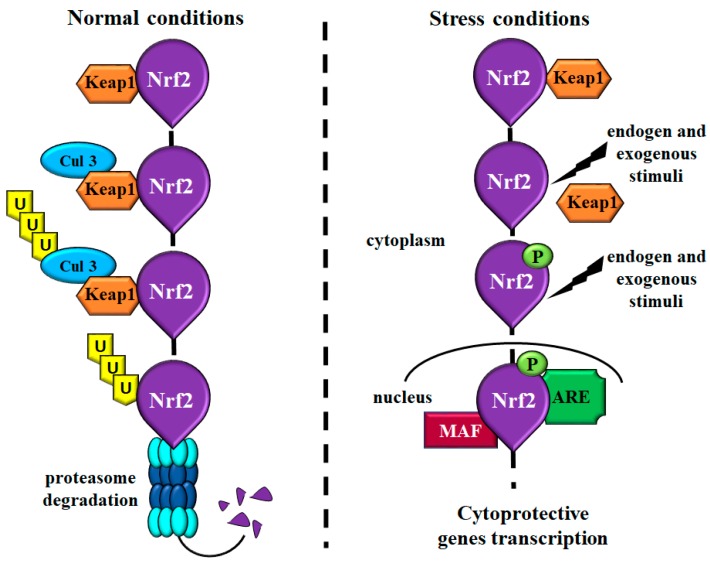
Schematic representation showing the main Nrf2 molecular mechanisms. Under normal condition, Nrf2 binds its inhibitor Keap1 that mediates the interaction with Cul3 leading to Nrf2 ubiquitination and proteasome degradation. These events provide a regulated modulation of Nrf2 levels in the cells. Under stress conditions and in response to different insults, Keap1 is inactivated, and Nrf2 is phosphorylated and translocated into the nucleus. In the nucleus, Nrf2 binds MAF proteins and, consequently, ARE sequences inducing the transcription of cytoprotective genes. Abbreviations: Cul3 = Cul3-based E3-ubiquitin ligase; Keap1 = Kelch-like ECH-associated protein 1; Nrf2 = nuclear factor-erythroid 2-related factor; MAF = musculoaponeurotic fibrosarcoma proteins; ARE = antioxidant responsive element; P = phosphorylated.

In 1994, Moi et al. identified Nrf2 as a transcription factor of beta-globin gene and, thereafter, several studies have revealed its key role in the regulation of many detoxifying enzymes, appearing to be a main player of the cytoprotective response against oxidative stress [26,27,28]. Nrf2 is a member of the cap’n’collar subfamily of basic region leucine zipper transcription factors that together with Nrf1, Nrf3, and NF-E2 p45 subunit, is able to regulate gene expression [22,24]. Under homeostatic conditions, Nrf2 transcriptional activity is mediated by the binding with its inhibitor Kelch-like ECH-associated protein 1 (Keap1) which mediates the interaction between Nrf2 and Cul3-based E3-ubiquitin ligase complex, leading to continuous ubiquitination and proteasome degradation of Nrf2. This mechanism ensures constant levels of Nrf2 in the cells (Figure 1) [22,25]. Instead, in response to repetitive endogens and exogenous stimuli, Keap1 inhibition fails and Nrf2 translocate from the cytoplasm to the nucleus where dimerizes with members of the musculoaponeurotic fibrosarcoma proteins (MAF). The heterodimer recognizes a specific sequence known as the antioxidant responsive element (ARE), situated in the promoter region of Nrf-2 target genes, and activates their transcription (Figure 1).

Initially, ARE was described as a regulatory element for a limited number of genes such as NAD(P)H dehydrogenase-(quinone 1) and glutathione transferase, but new functions of Nrf2 are constantly arising [29,30,31,32,33,34,35].

Currently, it is well known that there are several proteins encoded by ARE genes, including those involved in drug and xenobiotic detoxification and in oxidative stress response. In cellular redox homeostasis, Nrf2 can regulate critical functions such as biosynthesis of glutathione (GSH), thioredoxin (TXN), in addition to the regeneration of nicotinamide adenine dinucleotide phosphate oxidase (NADPH) and the production of ROS. Nrf2 could have a protective role in many autoimmune diseases (i.e., vitiligo, asthma, multiple sclerosis, and systemic lupus erythematosus) since an increased production of reactive immunogenic macromolecules intermediates in these disorders seems closely linked to the loss of several phase II detoxification enzymes regulated by the activation of Nrf2 genes [23,36].

Additionally, Nrf2/ARE signaling participates in heme metabolism, cellular redox homeostasis, the control of inflammatory responses, tissue remodeling, fibrosis, carcinogenesis, and metastasis [37,38].

## 3. Role of Nrf2 in Inflammation and Fibrosis in Various Organs 

Several studies have demonstrated that Keap1/Nrf2 signaling plays a pivotal role in the attenuation of both acute and chronic inflammation in several diseases (i.e., rheumatoid arthritis, asthma, emphysema, gastritis, and atherosclerosis) through its ability to normalize mitochondrial function, restore redox homeostasis, and suppress the production of inflammatory mediators [39,40,41,42,43,44].

Particularly, in an early phase of the inflammatory process, the Nrf2/ARE pathway seems to exert an inhibitory effect in the production of pro-inflammatory molecules such as cytokines, chemokines, cell adhesion proteins, MMPs, cyclooxygenase-2 (COX-2), and prostaglandins [45,46,47,48,49,50].

Due to the intimate link between inflammation and fibrosis, several studies have analyzed the potential role of Nrf2 to mitigate the release of several inflammatory molecules involved in abnormal collagen syntheses and deposition [51,52,53,54,55,56,57,58] (Table 1). 

## 4. Role of Nrf2 Signaling in Intestinal Inflammation and Fibrosis

After an injury, the organism activates a sequence of events to maintain the integrity of tissue morphology and functionality: (1) recruitment of inflammatory cells, (2) release of fibrogenic cytokines and, lastly, (3) activation of ECM-depositing cells [59].

Inflammation is the first biological response to adverse stimuli, such as pathogens (i.e., bacteria and viruses) and external damage (i.e., tissue scrapes and effects of chemicals and radiations) to limit and sometimes to eliminate the causes of cellular injuries. According to the type of cells involved and the injury duration, the inflammatory process can be classified as acute (rapidly and self-limiting response) or chronic (response prolonged for weeks, months or, in a specific scenario, for a lifetime). Protracted insults can lead to abnormal deposition of ECM proteins and consequently to a progressive pathological condition such as fibrosis. However, although several studies have tried to clarify the relationship between inflammation and fibrosis in IBD, the precise pathophysiological mechanism is still unknown and no effective drugs, as alternative to surgery, are available when stenosis has developed. In an early phase, inflammatory cells are recruited into the injury site to restore organ integrity and function. However, the inflammatory response protraction over time becomes chronic, leading to myofibroblast activation and uncontrollable ECM deposition and fibrosis [1,4,6]. It is well known that the key regulator of intestinal fibrosis is TGF-β, which exerts its function through its canonical (Smads) and non-canonical (i.e., mitogen-activated protein kinase (MAPK), sphingosine 1-phosphate, and mammalian target of rapamycin) pathways [60,61].

Moreover, recent findings have highlighted other crucial molecules able to interact with TGF-β and involved in the development of intestinal fibrosis, such as Nrf2 and its downstream genes (Table 2).

### 4.1. Nrf2 in Experimental Models of Colitis Induced in Transgenic Mice

In 2006, for the first time, Khor et al. showed in an experimental dextran sulfate sodium (DSS)-induced colitis model that Nrf2 knockout (KO) mice (Nrf2^−/−^) were more susceptible to acquiring histological features of colitis (such as a short length of the colon and rectal bleeding) compared to Nrf2 wild-type (WT) mice (Nrf2^+/+^). In the same study, overexpression of many antioxidant and phase II detoxifying enzymes (heme oxygenase-1, UDP-glucuronosyltransferase 1A1, and glutathione *S*-transferase Mu-1) was associated with an increase in Nrf2 levels in DSS WT mice compared to KO mice. Depletion of Nrf2 reduced the mRNA and protein expression of these enzymes and contributed to an increased level of pro-inflammatory mediators, supporting the hypothesis that Nrf2 plays a significant role in colonic damage [62]. 

Remarkably, the role of the Nrf2/inflammation axis in the progression of DSS-induced colitis has been demonstrated by genetic mouse experiments. In fact, Nrf2^−/−^ KO mice challenged by DSS presented more severe histopathological signs of colitis, showing a high expression of COX-2, HO-1, and γ-glutamylcysteine synthetase (γ-GCS) compared to WT controls. On the other side, DSS-induced colitis in COX-2^−/−^ KO mice was less severe compared to WT control mice, showing a decrease of heme oxygenase-1 (HO-1) and increase of NADPH quinone oxidoreductase-1 (NQO-1), demonstrating the tight association between inflammation and the antioxidant system [63].

Interestingly, in the same year, Li et al. found that high expression of Nrf2 and HO-1 were correlated with the level of mitogen-activated protein kinase phosphatase-1 (Mkp-1), not only in the colon of mice with DSS-induced colitis but even in colorectal biopsies of patients affected by UC and CD. Moreover, Mkp-1^−/−^ mice were more sensitive to DSS-induced colitis, showing severe signs of inflammation. The authors concluded that Mkp-1 and Nrf2/HO-1 create a protective function against inflammation [64].

It is known that during an inflammatory process, a range of damage-sensing receptors—including the inflammasome multiprotein complexes NOD-like receptor (NLR) family—are activated. Among these receptors, the most studied is NLPR3 and its dysregulation seems to contribute to IBD development [65]. In DSS-induced colitis using NLPR3^−/−^ KO mice, Wang et al. analyzed the anti-inflammatory effect of a novel Nrf2/ARE inducer, called compound 1. This molecule was able to reduce colorectal inflammation in DSS-induced colitis in NLPR3^+/+^ WT mice while it did not exert anti-inflammatory effects in DSS-induced colitis in NLPR3^−/−^ KO animals. This evidence highlighted that the protective effect of compound 1 was due to its inhibitory action on NLRP3 inflammasome and, concomitantly, Nrf2 activation [65].

### 4.2. Nrf2 Inhibition in Experimental Models of Colitis 

Several studies revealed that heme oxygenase, an important enzyme regulated by Nrf2, ameliorated inflammation in many experimental models [66,67,68,69] although its specific role in IBD remains unclear. 

To clarify this mechanism, Wang et al. analyzed the HO-1 involvement on trinitrobenzene sulfonic acid (TNBS)-induced colitis in mice. HO activity and HO-1 gene expression were significantly higher in mice receiving TNBS compared to controls. Administration of mesoporphyrin (SnMP), an HO inhibitor, potentiated the colonic damage along with a reduction in HO-1 activity. Furthermore, the reduction of HO-1 expression by SnMP also enhanced ROS and inducible nitric oxide synthase (iNOS) expression, both of which were dramatically increased after the TNBS enema. These results indicate a protective role of HO-1 and Nrf2 in TNBS-induced colitis by decreasing free radical production and inhibiting iNOS expression in colonic mucosa [70].

More recent investigations conducted both in vivo (by DSS-induced colitis as an experimental model of IBD) and in vitro (by colonic cells NCM460) revealed that CPUY192018, an inhibitor of the Keap1–Nrf2 interaction, reduced the expression of inflammatory cytokines and exerted a cytoprotective effect. These data suggested that molecules able to regulate Keap1–Nrf2–ARE signaling alleviated features of experimental colitis and could represent a new therapeutic strategy for IBD [71]. 

### 4.3. Nrf2 Activation in Experimental Models of Colitis

In an in vivo study of acetic acid-induced colitis in rats, Yalniz et al. demonstrated the ability of nadroparin sodium to prevent and attenuate the expression of nuclear factor kappa-light-chain-enhancer of activated B cells (NF-kB). The authors demonstrated that anti-inflammatory and antioxidative effects of nadroparin were mediated by Nrf2/HO-1/NF-kB pathways, showing that Nrf2/ARE signaling was involved in intestinal inflammatory processes [72]. 

In DSS-induced colitis, Liu et al. analyzed the effects of dimethyl fumarate (DMF), an immunomodulatory and anti-inflammatory drug currently used for relapsing forms of multiple sclerosis. The authors showed that DMF administration attenuated signs of DSS damage (i.e., body weight loss, colon length reduction), activated Nrf2 and its downstream genes, and inhibited pro-inflammatory cytokines and the NLRP3 inflammasome. Due to the ability of DMF to interfere with Nrf2 signaling, these results demonstrated a potential use of this drug also in the treatment of colitis [73]. 

Recently, in a mouse model of DSS-induced colitis, Yang et al. evaluated the impact of hyperoside (Hyp), a flavonoid with anti-inflammatory, antiapoptotic, and antioxidant proprieties. The results showed that Hyp reduced the expression of pro-inflammatory cytokines such as TNF-α, interleukin-6, COX-2, and NF-kB and increased the levels of anti-inflammatory cytokines such as interleukin-10 [74]. Additionally, the levels of pro-apoptotic proteins such as caspase-3 and Bax were reduced while antiapoptotic proteins, such as Bcl2, were overexpressed. The study demonstrated that Hyp ameliorates colitis by inducing expression of Nrf2 and its target genes. This effect was probably due to the ability of this substance to attenuate the inflammatory process and apoptosis through the activation of Nrf2 signaling [74]. 

Park et al. demonstrated the anti-inflammatory and antioxidative ability of a natural herb known as *Perilla frutescens* (PF). In DSS-induced colitis in mice, PF administration improved the features of colitis and reduced the expression of several pro-inflammatory molecules. PF was also able to inhibit the activation of NF-kB and STAT3 and, at the same time, to increase Nrf2 and HO-1 in the colon, displaying the protective effect of Nrf2 [75].

Another molecular partner of Nrf2 has been revealed by Jing et al. The study demonstrated that the features of colitis in DSS-treated rats were improved by administration of berberine, an alkaloid extracted from *Berberis* species. Berberine upregulated Nrf2 expression and increased P-glycoprotein (P-gp) both in vivo and in vitro in Caco-2 cells. Nrf2 silencing in Caco-2 cells abolished the upregulation of P-gp and, concomitantly, the beneficial effect of berberine [76]. Similar protective effects, through activation of Nrf2 signaling in DSS-induced colitis in mice, were described for luteolin, oligonol, and sinomenine, which were able to activate also the Nrf2-downstream partner NQO-1 [77,78,79]. Particularly, Li et al. revealed that luteolin was able to mitigate signs of fibrosis as well as the expression of inflammatory mediators (iNOS, TNF-α, IL-6) by activating Nrf2 and its downstream genes (HO-1 and NQO-1) [77]. Comparable results were found with the oligonol. This compound, by increasing Nrf2 action and the activation of HO-1 and NQO-1, leads to a decrease of IL-1, IL-6, TNF-α, NF-kB, c-Fos, and c-Jun [78]. In another study, Zhuo et al. highlighted the ability of sinomenine to improve DSS-induced colitis, interfering with the Nrf2/NQO-1 signaling and reducing the levels of pro-inflammatory and pro-fibrotic molecules (TNF-α, IL-6, and iNOS) [79]. Similarly, Gao et al. showed the protective role of the LL202, a synthetic flavonoid derivate, which, especially in macrophages, stimulates the Nrf2/HO-1 pathway in both TNBS and DSS-induced colitis [80]. This study also revealed that this natural compound reduced the expression of pro-inflammatory molecules through the activation of Nrf2/HO-1 signaling, amplifying the Nrf2 antioxidant effect [80]. 

Saber and colleagues explored the effect of olmesartan, an angiotensin II receptor type 1 blocker, in acetic acid-induced colitis in rats. The drug acted as an Nrf2 activator, NF-kB inhibitor, and apoptosis inhibitor [81]. Tussilagone, a molecule able to inhibit NF-κB activation and to induce Nrf2, reduced the main markers of murine colitis induced by DSS (i.e., TNF-α and IL-6) suggesting its potential role in the treatment of colitis [82].

Also, plants typical of Mediterranean scrubs, such as rosemary (*Rosmarinus officinalis*) and common sage (*Salvia officinalis*) and synthesized compounds such as carnosol [83] and carnosic acid [84] showed antioxidant and protective effects in both in vitro (colon cells line) and in vivo (DSS-induced colitis in mice) studies. Both carnosol and carnosic acid increased Nrf2 expression.

Sangaraju et al. demonstrated that the phytochemical galangin (GAL) was able to ameliorate DSS-induced colitis in BALB/c mice. GAL administration in DSS colitis in mice induced an increased level of anti-inflammatory cytokines (IL-10) and decreased levels of pro-inflammatory mediators (TNF, IL-6, and iNOS). The study also indicated a protective effect of GAL against intestinal fibrosis by the activation of HO-1 and, consequently, of Nrf2 [85].

A recent paper supported the role of Nrf2 in intestinal fibrosis through the evaluation of apocynin, an NADPH oxidase inhibitor, in DSS-induced colitis in mice. In fact, the recovery of colon length and weight, as well as a decrease in the number of inflammatory foci, was observed in DSS mice upon treatment with apocynin. Moreover, the expression of iNOS, COX-2, TNF-α, and monocyte chemoattractant protein-1 (MCP-1) were decreased in DSS mice treated with apocynin. In the same group, Nrf2 and HO-1 were significantly activated, exerting a protective role [86]. 

The interplay between colitis and oxidative stress has been reported, with a tight correlation between ROS production and the upregulation of Nrf2. A diet low in methionine (an inducer of ROS) reduced fibrotic scores and the inflammatory profile in DSS-induced colitis in mice compared to DSS mice fed with a regular diet [87]. The beneficial effect of natural compounds in ROS inhibition during the progression of colitis was also demonstrated with the use of catechins, of which legumes and tea are rich. These phytochemicals increased the expression of antioxidant molecules interacting with several pathways, including NF-κB, MAPKs, STAT1/3, and of Nrf2, and also modulated the intestinal flora [88]. Comparable effects have been highlighted for licochalcone A, a chalcone isolated from licorice root widely recognized in traditional Chinese medicine. Licochalcone A was able to reduce oxidative stress and inflammation downregulating NF-kB and upregulating Nrf2 [89]. In fact, in DSS-induced colitis in mice, licochalcone administration reduced the histological scores of colitis and the levels of pro-inflammatory cytokines [89].

The Nrf2 involvement in experimental DSS colitis was also investigated by Li et al. and demonstrated the potential therapeutic role of ZnO nanoparticle (ZnONP) in the treatment of colitis in mice. The data highlighted that ZnONP suppressed ROS and pro-inflammatory cytokine production and activated Nrf2 signaling which was then able to carry out its antioxidant and anti-inflammatory functions. Furthermore, unlike 5-aminosalicylic acid (5-ASA), ZnONP was able to restore the changes in colonic bacteria occurring during colonic injuries and to maintain gut homeostasis. The authors suggested that ZnONP combined with 5-ASA, currently used in IBD treatment, could be a novel therapeutic agent in the treatment of colitis [90].

In TNBS-induced colitis model, other investigators demonstrated that the main markers of fibrosis (α-smooth muscle actin, collagen I, TIMP-1, and TGF-β1/Smad signaling) and ROS levels were suppressed by *tert*-butylhydroquinone (tBHQ), an agonist of Nrf2 [91]. The authors revealed that TNBS-induced intestinal fibrosis was reversed by tBHQ administration through inhibition of the ROS-dependent TGF-β1/Smad pathway [21].

**Figure 2:**
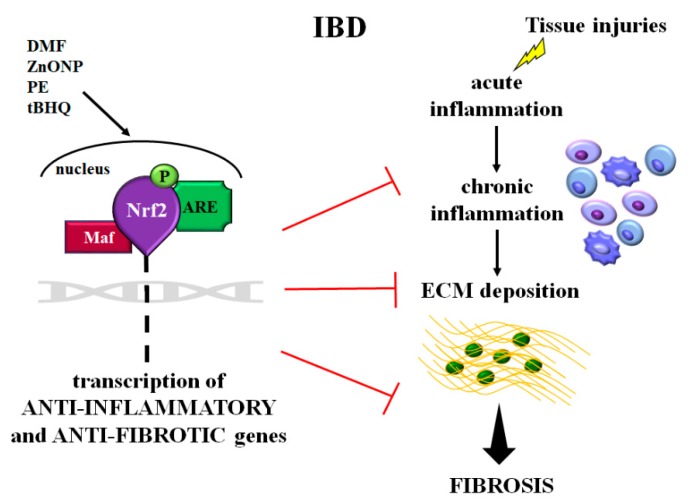
Schematic representation of the role of Nrf2 in intestinal inflammation and fibrosis occurring in inflammatory bowel disease (IBD). After tissue injuries, the cell’s response is characterized by a cascade of events: acute inflammation, chronic inflammation, and ECM proteins deposition leading to fibrosis. In these pathological events, Nrf2 is activated and then induces the transcription of the anti-inflammatory and antifibrotic genes. Molecules such as DMF, ZnONP, PE, and tBHQ, improving Nrf2 signaling, could be a new therapeutic strategy for intestinal inflammation and fibrosis. Abbreviations: ECM = extracellular matrix; Nrf2 = muclear factor-erythroid 2-related factor; Maf = musculoaponeurotic fibrosarcoma proteins; ARE = antioxidant responsive element; P = phosphorylated; DMF = dimethyl fumarate; ZnONP = ZnO nanoparticle; PE = *Perilla frutescens*; tBHQ = *tert*-butylhydroquinone.

All these findings revealed that, as occurs in other organs, Nrf2/ARE signaling acts both directly and indirectly (manly cooperating with TGF-β/Smad pathway) to reduce inflammatory and fibrotic processes, and could be a novel effective therapeutic target in IBD [21,73,75,90] (Figure 2).

## 5. Role of Nrf2 in the Cancer of Various Organs

The Nrf2 pathway certainly represents one of the main relevant pathways in cell defense and survival signaling, and its involvement in cancer has been extensively investigated in many organs. Data from the literature highlights that this multifunctional molecule exerts a double and controversial role in cancer development [12,13,14,17,18]. 

On the one hand, in an early stage of the disease, Nrf2 exerts a protective role against chemical-induced carcinogenesis through its ability to reduce ROS accumulation and, consequently, DNA injuries in cells [12,91,92,93,94,95] (Table 3). On the other hand, in the late stage of tumor progression, there is what is called “the dark side” of Nrf2, since it seems to be involved in cancer proliferation, and in chemo- and radioresistance [13,14,17,96,97,98,99,100,101,102] (Table 3).

## 6. Role of Nrf2 in IBD-Associated Colorectal Cancer 

Colorectal cancer (CRC) is the third most common cancer in men and the second in women worldwide and is the second cause of cancer death in a number of Western countries [103,104,105,106]. CRC is a highly common malignancy in European countries and throughout the world; it has been estimated that 1.13 million new CRC cases are diagnosed every year and that CRC causes about 694,000 deaths per year [106]. CRC is sporadic in 90% of patients; in <10%, it is inherited or is a complication of IBD [107,108].

CRC is linked to a wide range of risk factors such as diet, genetic and epigenetic alteration, immune response, oxidative stress, intestinal microbiota modification, as well as intestinal chronic inflammation [109,110,111,112,113]. Several clinical studies showed that 1 in 6 people affected by ulcerative colitis die of CRC and that chronic inflammation occurring during IBD may increase the risk of CRC development [114,115,116,117,118,119]. Inflammation activates a complex network of cytokines, chemokines, and inflammatory cells (i.e., neutrophils, macrophage, and lymphocytes) generating an environment enriched in ROS and NOS that represents the main factor which contributes to neoplastic transformation in IBD [112,118,120,121,122].

**Figure 3:**
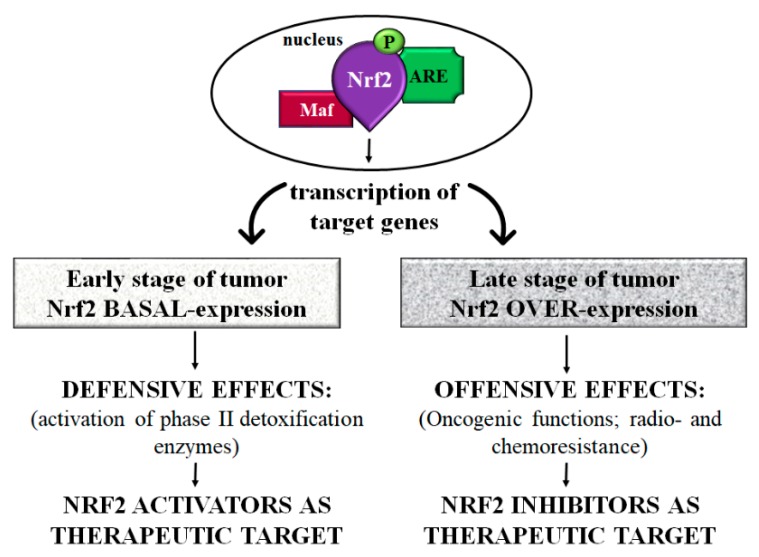
Scheme of Nrf2 controversial role in colorectal cancer. In the early stage of the tumor, Nrf2 exerts protective effects through the activation of phase II detoxification enzymes. In this step, Nrf2 activators maintaining constant levels of Nrf2 could act as a preventive agent in the development and progression of CRC. On the contrary, at the late stage of the tumor, there is an overexpression of Nrf2 that exerts negative effects characterized by tumor progression and metastasis in addition to radio- and chemoresistance. In this context, using Nrf2 inhibitors to regulate Nrf2 levels might represent a potential adjuvant treatment in CRC. Abbreviations: Nrf2 = nuclear factor-erythroid 2-related factor; Maf = musculoaponeurotic fibrosarcoma proteins; ARE = antioxidant responsive element; P = Phosphorylated.

As previously mentioned, several studies showed that Nrf2 plays a “double game” in tumors since, in the first stage of the disease, it exerts a protective role, while in the late stage it may have negative effects in the carcinogenetic process of many organs, including the gut [12,123] (Figure 3) (Table 4). 

### 6.1. Nrf2 Plays a “Protective Effect” in the Early Stage of Colorectal Cancer

In 2008, Khor et al. used an experimental model of colitis associated with azoxymethane (AOM)/DSS-induced cancer to show that CRC risk is higher in Nrf2 knockout mice. The study highlighted that signs of colitis, as well as the number of tumors per mouse, were increased in Nrf2^−/−^ KO mice compared to wild-type animals. These data demonstrated that Nrf2 was involved not only in protection against inflammation but also against inflammation-associated CRC [12]. 

Since endogenous estrogen in females has been shown to protect against the development of colon cancer, Song et al. demonstrated that exogenous estrogen replacement in ovariectomized mice showed a protective effect against AOM/DSS-induced colitis and carcinogenesis. The authors revealed that the action of estrogens can be ascribed to a network of estrogen receptors and NF-κB and Nrf2 pathways [124]. 

Many studies have shown that increased risk of injuries in different organs is correlated to a single-nucleotide polymorphism (SNP) in the promoter region of Nrf2 [43,125,126,127,128]. To clarify the link between Nrf2 and tumor risk, Yokoo and colleagues exposed mice to potassium bromate (KBrO_3_) to induce neoplastic proliferation comparable to human hereditary colorectal cancer, demonstrating that patients with Nrf2 SNP polymorphisms were more susceptible to colorectal cancer risk. The study showed that the incidence of preneoplastic and neoplastic damage in addition to the overexpression of Nrf2-regulated genes (COX2, proliferating cell nuclear antigen) was significantly higher in Nrf2-deficient mice compared to wild-type animals after a high dose of KBrO_3_ injection [129]. 

Jang et al. evaluated the protective effect of simvastatin, a synthetic derivative from the fermentation of *Aspergillus terreus*, on the expression of Nrf2 in two lines of human colon cancer cells. The study showed that simvastatin induced overexpression of antioxidant enzymes (HO-1, NQO-1, and glutamate–cysteine ligase catalytic subunit) and of Nrf2 in addition to its nuclear translocation. Additionally, using PI3K/Akt and ERK inhibitors, the authors demonstrated the ability of simvastatin in Nrf2 induction in colon cancer cells was mediated by these two pathways [130]. 

Considering the protective role of Nrf2, the identification of molecules able to sustain constant levels of this factor in an early stage of CRC could be a potential way to prevent cancer development and progression (Figure 3). For this reason, nowadays, many Nrf2 activators of both natural (i.e., sulforaphane, curcumin, resveratrol, kahweol, lycopene, carnosol) [131,132,133,134,135,136] and synthetic (i.e., oltipraz, dimethyl fumarate) origin are constantly tested in order to discover new effective treatments for CRC [137,138,139,140,141]. Indeed, these compounds proved to be able to protect cells from carcinogenic insults by the activation of the phase II detoxification enzymes and, consequently, to increase the Nrf2 cytoprotective response.

The most well-investigated natural molecule was sulforaphane, that if consumed in high doses, reduced the risk of cancer development in many organs, including colon [93,94,134,135,136]. The phenolic compounds epigallocatechin-3-gallate (EGCG), the main active catechin present in green tea, seems to exert antioxidant, anti-inflammatory, and chemopreventive functions in cancer. In in vitro experimental studies using colon cancer cells (Caco-2), EGCG was able to induce Nrf2 through ERK1/2 activation and Akt phosphorylation. The data suggest that due to its anti-inflammatory properties and its ability to interfere with Nrf2 signaling, EGCG could be an effective drug to prevent colorectal cancer development [131]. Trivedi et al. demonstrated that melatonin (MEL) exerted beneficial effects in colitis-associated colon carcinogenesis (CACC). The study showed that MEL was able to decrease autophagy, increase p62 levels, and activate Nrf2, leading to an enhancement of expression of various antioxidant enzymes. The results highlighted that MEL, through its ability to modulate Nrf2 signaling, attenuated the progression of CACC in mice [132]. In a study conducted by Zuo, luteolin (LUT), a dietary flavone, suppressed colorectal carcinogenesis, acting through Nrf2 epigenetic modifications [133]. The same molecule was tested by Kang et al., which revealed that LUT was able to increase the mRNA expression of Nrf2 by DNA demethylase and by the interaction between Nrf2 and p53, exerting a pro-apoptotic effect [142]. The effect of Shaoyao decoction (SYD), another natural compound, was tested by Wang et al. in AOM/DSS-induced CRC in mice. The authors demonstrated that SYD induced a reduction of oxidative stress and inflammation, activating Keap1–Nrf2–ARE signaling. These findings suggest that SYD plays an antioxidant effect and consequently prevents colitis-associated CRC [143]. In a recent paper, *Sageretia thea* has been reported as a potential drug of colorectal cancer. In fact, Kim et al. demonstrated that *S. thea* decreased the viability of cancer cells and increased HO-1 expression, activating Nrf2 [144]. 

Among the synthetic molecules, oltipraz is one of the most studied. In many organs, it was shown to be an effective inhibitor of chemically induced carcinogenesis processes [96,137,138,139,140,145,146,147,148,149]. However, in the bowel, its role is controversial, since chronic doses of this drug seems to increase colorectal cancer risk [142].

### 6.2. Nrf2 Plays an “Offensive Effect” in Late-Stage Colorectal Cancer

C–X–C chemokine receptor type 4 (CXCR4) and Nrf2 signaling (both normally activated during the cellular response in cancer) appears to be associated with clinical features in patients affected by CRC. High levels of CXCR4 and Nrf2 were correlated with increased tumor recurrence and lymph node and distance metastasis, suggesting that anomalous levels of these two molecules are involved in CRC progression [150].

Multidrug resistance (MDR) represents one of the main problems in CRC treatment and the mechanisms underlying this process are still unclear. In an in vitro study, Zhao et al. analyzed epigenetic modification of the Nrf2 gene to clarify the drug resistance mechanisms observed in colon cancer cells treated with 5-fluorouracil (5-FU). High levels of Nrf2 and its nuclear translocation accompanied the increased HO-1 and ROS levels found in resistant CRC cells (SNU-C5R) compared to controls (SNU-C5). These results suggest that overexpression of Nrf2 and an increase of HO-1 activity are involved in drug resistance occurring in colon cancer [151]. Zhang et al. demonstrated that curcumin was involved in MDR in colorectal cancer cell lines through a direct link with Nrf2. In fact, this compound, in combination with 5-FU, was able to induce apoptosis and reduce Nrf2 and Bcl-2/Bax expression, leading to a reversal effect on MDR in colon cancer [152]. Recently, Zhang et al. revealed, for the first time, the involvement of DUB3—an alternative Nrf2 deubiquitinating enzyme—in chemoresistance occurring in colorectal cancer. The authors demonstrated that DUB3 could be a new therapeutic target for the development of new drugs able to stabilize levels of Nrf2 and counteract its chemotherapy resistance effect [153].

Other studies have focused on the possible correlation between Nrf2 subcellular localization and the development and progression of colorectal cancer. In in vivo experiments, Lin et al. evaluated the expression of antioxidant enzymes (i.e., NQO-1 and HO-1) on colorectal tumor fragments showing that Nrf2 was exclusively expressed in the nucleus (nNrf2) in NQO-1- and HO-1-positive tumors, while it was retained in the cytoplasm (cNrf2) in NQO-1- and HO-1-negative tissues. Additionally, patients with cNrf2 tumors had a poorer outcome compared to those with nNrf2. In in vitro experiments, they demonstrated that cNrf2 increased tumor invasiveness through the upregulation of proteasome non-ATPase regulatory subunit 4 (PSMD4), a crucial enzyme in proteasome complex assembly. These data revealed Nrf2/PSMD4 involvement in colorectal cancer progression [154]. Further confirmation that cNrf2 acts as pro-oncogenic factor came from a study made by Cheng et al. They showed that cNrf2 conferred chemoresistance to 5-FU and oxaliplatin both in vitro (HCT116 cells) and in vivo (animal model of CRC). The resistance mechanism involved activation of PSMD4 which, in turn, enhanced Nrf2 export from the nucleus and, finally, the activation of NF-κB/AKT/β-catenin/ZEB1 cascades. These experimental observations were confirmed in colorectal cancer patients, in whom a higher prevalence of unfavorable chemotherapeutic response was noted in subjects with cNrf2- and PSMD4-positive tumors [155]. 

There is evidence that the antitumor effects of some compounds are related to their inhibition of Nrf2, while stimulation of tumor growth induced by certain substances is mediated by the activation of Nrf2 [156,157,158]. In a recent study, Thajmohammadi and colleagues demonstrated that stattic (a STAT3 inhibitor) represented an effective molecule to increase the cytotoxic effect of 5-FU in colon cancer cells. The study showed that the simultaneous administration of stattic and 5-FU increased the antitumor effect, decreasing Nrf2 and Bcl-2 levels and, at the same time, increasing the level of Bax in tumor cells. These findings revealed that stattic could be an effective treatment in colon cancer due to its pro-apoptotic function by modulating the Nrf2 expression [156]. Cernigliaro et al., in an in vitro study, reported the effect of ethanol (EtOH) administration on the survival of colon cancer cells. The authors revealed that EtOH increased the expression of antioxidant enzymes and induced Nrf2 nuclear translocation. These data supported the hypothesis that EtOH promotes progression and aggressiveness of colon cancer cells through the activation of Nrf2/HO-1 signaling [157]. In another study, it was reported that glyceollins (soybean-derived phytoalexins) are involved in the development of colon cancer. Using a xenograft BALB/c nude mouse model, the researchers showed that glyceollin administration induced tumor growth by activating Nrf2 signaling and inducing overexpression of HO-1 [158]. Taira et al. demonstrated the ability of marine peroxy sesquiterpenoids, an extract of *Sinularia* sp. coral, to exert an antitumor effect in human colon cancer cells, suppressing Nrf2-ARE signaling and inducing apoptosis in cancer cells [159]. This natural extract was able to inhibit antiapoptotic molecules (B-cell lymphoma-extra large (Bcl-xL) and phospho-Akt (p-Akt)) and to reduce HO-1, Nrf2, and its phosphorylated form in a colon cancer cell line (HCT116) [159].

In 2018, Sadeghi and colleagues tested the relationship between clinicopathological features of CRC and the expression of Nrf2, Keap1, and ATP binding cassette subfamily B member 1 (ABCB1) [160]. The study highlighted that Nrf2 and ABCB1 genes were overexpressed in CRC fragments compared to controls, showing Nrf2 involvement in CRC pathogenesis. On the contrary, the Nrf2 inhibitor Keap1 was significantly higher in controls with respect to the CRC group. These findings suggest that Nrf2 and P-gp (the main product of the ABCB1 gene) are implicated in CRC development and progression in addition to chemoresistance occurring in the tumor. Inhibitors of the Nrf2-ABCB1/P-gp axis could be useful in increasing the effectiveness of CRC chemotherapeutic drugs. 

As Nrf2 may play a central role in tumor progression and in chemo- and radioresistance, Nrf2 inhibitors have emerged as a promising adjuvant therapy to improve chemotherapeutic drug effectiveness (Figure 3). Several Nrf2 inhibitors, including plant extracts (as flavonoids and alkaloids) or synthetic molecules (as ARE expression modulator 1 and metformin), have also been tested in CRC [18,97,161,162]. Particularly, Yao and colleagues revealed, both in vivo and vitro experiments, that wogonin, a flavonoid-like chemical compound, suppress inflammation-associated colon carcinogenesis and cancer development. Wogonin inhibited cell proliferation and production of pro-inflammatory mediators and regulates expression of NF-jB and Nrf2 [163]. Recently, Evans et al. reported that primary CRC and metastatic samples showed high levels of Nrf2 compared to control group. Moreover, in the same study, brusatol (Nrf2 natural inhibitor) injection counteracted tumor growth, suggesting that Nrf2 could be a potential target in CRC treatment [164].

Among synthetic molecules, it was demonstrated that metformin reduced mRNA levels of Nrf2 and patients affected by CRC treated with metformin showed a better prognosis [18,165,166]. Henderson et al. reported that metformin administration improved the prognosis of patients with CRC by reducing tumor recurrences, metastasis, and deaths [165]. Sena et al. treated a colon cancer cell line (HT29) with different concentration of metformin, showing an increase in apoptosis and autophagy processes in treated compared to untreated cells. The study highlighted that the anticancer effects of metformin are correlated with Nrf2 inhibition [166]. All these data suggest that metformin could be an efficacious candidate for the treatment of CRC.

## 7. Conclusions 

Inflammatory bowel diseases, including Crohn’s disease and ulcerative colitis, represent a challenging clinical condition since they can lead to severe complications such as intestinal fibrosis and colorectal cancer which are currently manageable only through surgery.

Limiting IBD progression and the development of chronic complications like fibrosis and cancer represents an important milestone. Identifying the main molecules responsible for the development of IBD-associated intestinal fibrosis and cancer is crucial for the prevention of such severe complications. Nrf2, a transcriptional factor regulating many antioxidant genes, seems to be a good candidate, showing a defensive role against inflammation, fibrosis, and cancer in various organs, including the intestine. Several studies have shown that Nrf2 is directly and strongly involved in the intestinal fibrosis and colorectal cancer occurring in IBD. It emerges that the use of Nrf2 activators to maintain constant levels of this molecule in the first stage of many inflammatory diseases can limit their progression and complications such as fibrosis and cancer. On the other hand, as Nrf2 is overexpressed in advanced cancer, Nrf2 inhibitors may represent an effective therapeutic adjuvant leading to a significant reduction of radio- and chemoresistance. Nevertheless, future studies are needed to clarify the complex role of Nrf2 and the possibility to develop new drugs able to modulate the Nrf2 pathway and to potentiate its protective effect. This scenario could be a valid chance to limit the two main IBD chronic complications—intestinal fibrosis and cancer.

## Figures and Tables

**Table 1:** Studies assessing Nrf2 involvement in inflammation and fibrosis in various organs.

References	Organ	Nrf2 Action
Xu et al., 2008 [51]	Liver	Nrf2-deficient mice showed a decreased expression of cytoprotective proteins together with reduced development and progression of liver fibrosis compared to wild-type mice.
Chen et al., 2013 [54]		Upregulation of Nrf2 induced by glycyrrhetinic acid prevented CCl4-induced liver fibrosis in mice.
Prestigiacomo et al., 2018 [55]		Overexpression of the main markers of fibrosis (α-SMA, collagens, fibronectin) in Nrf2/Keap1 knockout mice compared to wild-type animals. The TGFβ-1/Smad pathway was able to mediate hepatic stellate cells activation and then fibrogenesis in mice with Nrf2 depletion.
Kikuchi et al., 2010 [53]	Lung	An increase of oxidative stress markers associated with a higher degree of inflammation and fibrosis was found in Nrf2^−/−^ KO mice compared to controls.
Liu et al., 2019 [57]		Costunolide suppressed the development of pulmonary fibrosis through its ability to inhibit NF-kB and to regulate TGF-β1/Smad2/Nrf2-NOX4 signaling.
Zhang et al., 2018 [56]	Kidney	Testosterone propionate attenuated renal fibrosis age-related through TGF-β1/Smad deletion and parallel Nrf2/ARE activation in aged rats.
Chen et al., 2019 [58]	Hearth	Irisin was able to play an antifibrotic effect via Nrf2 through the inhibition of ROS/TGFβ1/Smad2-3 signaling.

**Table 2:** Studies assessing Nrf2 involvement in intestinal inflammation and fibrosis.

References	Nrf2 protective effect
Khor et al., 2006 [62]	Nrf2^−/−^ KO mice were more susceptible to colitis compared to WT mice demonstrating the significant role played by Nrf2 in intestinal inflammation.
Lee et al., 2018 [63]	COX-2^−/−^ KO mice were protected to colitis progression, while Nrf2^−/−^ KO mice showed severe signs of colitis demonstrating the protective effect of Nrf2 in intestinal inflammation.
Li et al., 2018 [64]	Mkp-1^−/−^ mice were more sensitive to DSS insult. Mkp-1 correlated with Nrf2/HO-1 expression in intestinal colitis.
Wang et al., 2016 [65]	NLRP3 inhibition, concomitantly to Nrf2 activation, protected from DSS-induced colitis.
Wang et al., 2001 [70]	Mesoporphyrin, an HO-inhibitor, induced progression of colonic injuries in TNBS-induced colitis.
Lu et al., 2016 [71]	The inhibitor of Keap1/Nrf2 (CPUY192018) alleviated DSS-induced colitis in mice.
Yalniz et al., 2012 [72]	Nadroparin sodium exerted anti-inflammatory and antioxidative effects through Nrf2/HO-1/NF-kB signaling.
Liu et al., 2016 [73]	Dimethyl fumarate activated Nrf2 and inhibited NLRP3, attenuating signs of DSS-induced colitis.
Yang et al., 2017 [74]	Hyperoside, by increasing Nrf2, attenuated inflammation in DSS-induced colitis in mice.
Park et al., 2017 [75]	A natural compound, *Perilla frutescens*, increased Nrf2, decreased NF-kB, and improved features of DSS-induced colitis.
Jing et al., 2018 [76]	Berberine induced Nrf2 and P-gp both in vitro and in vivo, leading to an attenuation of colitis.
Li et al., 2016 [77]	Luteolin, through the activation of Nrf2, mitigates intestinal inflammation.
Kim et al., 2018 [78]	Oligonol activated Nrf2, exerting a protective effect in experimental colitis.
Zhou et al., 2018 [79]	Sinomenine induced Nrf2 and NQO-1 and ameliorated signs of colitis.
Gao et al., 2019 [80]	Synthetic flavonoid molecule LL202 showed a protective effect on colitis, stimulating Nrf2/HO-1 signaling.
Saber et al., 2019 [81]	Olmesartan blocking Angiotensin II receptor type 1 activated Nrf2 and mitigated colitis in acetic acid-induced colitis.
Cheon et al., 2018 [82]	Tussilagone inhibited NF-kB and induced Nrf2, reducing the main markers of colitis induced by DSS in mice.
Yan et al., 2018 [83]	Carnosol increased the expression of Nrf2 and its downstream genes leading to antioxidant effects.
Yang et al., 2017 [84]	Carnosic acid exerted a protective effect in DSS-induced colitis inducing overexpression of Nrf2.
Sangaraju et al., 2019 [85]	Galangin was able to increase HO-1 and to reduce pro-inflammatory cytokines expression in DSS-induced colitis.
Hwang et al., 2019 [86]	Apocynin activated the Nrf2/HO-1 pathway and decreased intestinal inflammation and fibrosis in DSS-induced colitis in mice.
Liu et al., 2017 [87]	Low methionine diet protected from the progression of colitis and fibrosis induced by DSS mice.
Fan et al., 2017 [88]	Catechins reduced ROS, increased Nrf2 levels, and modulate intestinal flora, exerting beneficial effects in colitis.
Liu et al., 2018 [89]	Licochalcone A, a molecule able to attenuate oxidative stress and inflammation by decreasing NF-kB and increasing Nrf2 expression.
Li et al., 2017 [90]	ZnONP activated Nrf2, suppressed ROS and pro-inflammatory cytokines, and preserved gut microbiota.
Guan et al., 2018 [21]	An agonist of Nrf2 (tBHQ) ameliorated markers of fibrosis in TNBS-induced chronic colitis.

**Table 3:** Studies illustrating positive (advantages) and negative (disadvantages) roles of Nrf2 in cancers of various organs.

**Advantages of Nrf2**		
**References**	**Tumor Site**	**Nrf2 Action**
Ramos-Gomez et al., 2003 [91] Fahey et al., 2002 [93]	Stomach	Benzo(a)pyrene-induced gastric neoplasia was reduced in Nrf2 knockout compared to wild-type mice. Sulforaphane blocked benzo(a)pyrene-evoked forestomach tumors in mice. This protection was abrogated in mice lacking the *nrf2* gene.
Iida K et al., 2004 [95]	Bladder	Increased incidence of bladder tumors was found in Nrf2^−/−^ knockout mice compared to wild-type animals following *N*-nitrosobutyl(4-hydroxybutyl)amine administration.
Xu et al., 2006 [94]	Skin	Higher incidence of skin tumors after 7,12-dimethylbenz(a)anthracene or 12-*O*-tetradecanoylphorbol-13-acetate administration was detected in Nrf2-deficient mice compared to wild-type mice.
**Disadvantages of Nrf2**		
**References**	**Tumor Site**	**Nrf2 Action**
Singh et al., 2006 [13] Shibata et al., 2008 [14] Zhang et al., 2010 [17] Kim et al., 2010 [96] Singh et al., 2016 [97]	Lung, gallbladder, esophagus, skin prostate, breast, head, neck, ovary, endometrium	Increased levels of Nrf2 expression leading to a progression of all these types of cancer and, consequently, to a poor prognosis.
Mitsuishi et al., 2012 [98]	Lung	High levels of Nrf2 accelerate cancer cell proliferation, inducing purine nucleotide and glutathione synthesis. Nrf2 directly activates metabolic genes under active PI3K-Akt signaling.
Satho et al., 2013 [99] Satho et al., 2016 [100]		Nrf2 prevents initiation but accelerates progression through the Kras signaling pathway during lung carcinogenesis.
Shim et al., 2009 [101]	Ovary	Activation of the Nrf2 pathway can participate in the acquisition of doxorubicin resistance in ovarian carcinoma cells.

**Table 4:** Studies showing “protective” and “offensive” effects of Nrf2 in colorectal cancer.

**Protective Effects of Nrf2**	
**References**	**Nrf2 Action**
Khor et al., 2008 [12]	Nrf2^−/−^ KO mice showed an increased susceptibility to colitis-associated colorectal cancer (CRC) compared to Nrf2^+/+^ WT mice.
Osburn et al., 2007 [92]	Nrf2-deficient mice upon DSS treatment showed an increased formation of colorectal aberrant crypt foci and cancer compared to wild-type mice.
Song et al., 2019 [124]	Estrogens exerted a protective effect in the development of colon cancer through estrogen receptor, and NF-kB and Nrf2 pathways.
Yokoo et al., 2016 [129]	Incidence of colorectal preneoplastic and neoplastic lesions was significantly higher in Nrf2-deficient mice.
Jang et al., 2016 [130]	Simvastatin was able to induce a protective effect inducing Nrf2 overexpression and cooperating with PI3K/Akt and Erk pathways.
Kou et al., 2013 [131]	EGCG induced Nrf2, exerting antioxidant, anti-inflammatory, and antineoplastic functions.
Trivedi et al., 2016 [132]	Melatonin activated Nrf2 and p62 and decreased autophagy. In colitis-associated colorectal carcinogenesis, melatonin reduced the progression of colorectal cancer.
Zuo et al., 2018 [133]	Luteolin is an epigenetic modulator of Nrf2 and suppressed carcinogenesis in colon cancer cells.
Kang et al., 2019 [142]	Luteolin increased Nrf2 expression, showed an interaction between Nrf2 and p53, and induced apoptosis.
Wang et al., 2019 [143]	SYD reduced oxidative stress and inflammation in an AOM/DSS mouse model, activating Keap1–Nrf2–ARE signaling and preventing colitis-associated CRC.
Kim et al., 2019 [144]	*Sageretia thea* induced the Nrf2/HO1 pathway and decreased viability of colorectal cancer cells.
**Offensive Effects of Nrf2**	
**References**	**Nrf2 Action**
Hu et al., 2013 [150]	Nrf2 and CXCR4 were correlated with lymph node and distant metastasis in CRC patients.
Zhao et al., 2015 [151]	Nuclear translocation of Nrf2 and high expression of HO-1 and ROS were found in colorectal carcinoma cell line resistant to 5-Fu.
Zhang et al., 2019 [152]	Deubiquitination of Nrf2 by DUB3 stabilized Nrf2–Keap1 complex promoting chemoresistance in colorectal cancer cells.
Zhang et al., 2018 [153]	Curcumin corroborated 5-Fu effect in colon cancer reducing Nrf2 and Bcl2-Bax expression and inducing apoptosis.
Lin et al., 2016 [154]	Cytoplasmic Nrf2-positive tumors had a poor outcome, increasing tumor invasiveness by upregulation of PSMD4.
Cheng et al., 2018 [155]	Cytoplasmic Nrf2 conferred chemoresistance to 5-Fu and oxaliplatin, in both in vitro and in vivo studies.
Tajmohammadi et al., 2019 [156]	STAT3 inhibitor improved 5-Fu effect in colon cancer cells, decreasing Nrf2 and Bcl2.
Cernigliaro et al., 2019 [157]	Ethanol increased Nrf2 nuclear translocation and activated Nrf2/HO-1 signaling, favoring survival and aggressiveness of CRC cells.
Jeong et al., 2019 [158]	Glyceollins activated Nrf2 and HO-1, promoting tumor growth in a BALB/c xenograft.
Taira et al., 2018 [159]	Coral extract suppressed Nrf2 signaling, inducing apoptosis in human colon cancer cells.
Kensler et al., 2005 [134]	Nrf2 induced ABCB1 expression and consequent chemoresistance in CRC.
